# Sensitivity to value-driven attention is predicted by how we learn from value

**DOI:** 10.3758/s13423-016-1106-6

**Published:** 2016-06-29

**Authors:** Sara Jahfari, Jan Theeuwes

**Affiliations:** Department of Cognitive Psychology, VU University Amsterdam, Amsterdam, Netherlands

**Keywords:** Visual attention, Reinforcement learning, Reward, Q-learning, Bayesian hierarchical modeling

## Abstract

Reward learning is known to influence the automatic capture of attention. This study examined how the rate of learning, after high- or low-value reward outcomes, can influence future transfers into *value-driven attentional capture*. Participants performed an instrumental learning task that was directly followed by an attentional capture task. A hierarchical Bayesian reinforcement model was used to infer individual differences in learning from high or low reward. Results showed a strong relationship between high-reward learning rates (or the weight that is put on learning after a high reward) and the magnitude of attentional capture with high-reward colors. Individual differences in learning from high or low rewards were further related to performance differences when high- or low-value distractors were present. These findings provide novel insight into the development of *value-driven attentional capture* by showing how information updating after desired or undesired outcomes can influence future deployments of automatic attention.

It is well known that attention can be captured automatically by salience, past experiences, and learned reward associations (Awh, Belopolsky, & Theeuwes, 2012). Reward-driven attentional biases are known to develop as a consequence of implicit stimulus–reward associations learned in the past (Anderson, Laurent, & Yantis, 2011; Chelazzi, Perlato, Santandrea, & Della Libera, 2013; Gottlieb, Hayhoe, Hikosaka, & Rangel, 2014), are known to scale with the learned value of past rewards (Anderson & Yantis, 2012; Della Libera & Chelazzi, 2006), and increase stimulus saliency for future decisions (Failing & Theeuwes, 2014; Ikeda & Hikosaka, 2003; Kiss, Driver, & Eimer, 2009; Schiffer, Muller, Yeung, & Waszak, 2014). However, despite the progress in understanding the consequences of reward on attention and saliency, the underlying mechanisms by which reward associations come to shape automatic value-driven attention remain largely elusive.

Reward associations are learned through past experiences where an event (e.g., choosing a stimulus) is linked to a probabilistic outcome. Influential learning theories suggest that when an organism receives new information (e.g., choice outcome), current beliefs are updated in proportion to the difference between expected and actual outcomes (termed prediction error, δ). Notably, the degree by which prediction errors come to change stimulus–reward associations is determined by an additional factor termed learning rate, α (Daw, 2011; Sutton & Barto, 1998; Watkins & Dayan, 1992). Learning rates describe the rate by which new information replaces old and are fundamental to adaptive behavior. Higher learning rates result in greater trial-to-trial belief adjustments after a single instance of feedback and are linked to dopamine levels within the striatum (Frank, Moustafa, Haughey, Curran, & Hutchison, 2007) or activity changes within the anterior cingulate cortex (Behrens, Woolrich, Walton, & Rushworth, 2007); a region known to evaluate prediction errors and choice difficulty (cf. Brown & Braver, 2005; Shenhav, Straccia, Cohen, & Botvinick, 2014).

We examined whether learning rates have a direct impact on the development of value-driven attentional capture. Studies focusing on the interaction between value and capture show differential effects for high- and low-value rewards. Especially when learning is based on low value, subsequent tests assessing capture show smaller or no effects (Anderson & Yantis, 2012; Della Libera & Chelazzi, 2006). Reinforcement studies provide a possible explanation for this effect: cognitive models that are used to predict trial-to-trial learning behavior generally show higher rates for positive outcomes relative to negatives ones (Frank et al., 2007; Kahnt et al., 2009). Hence, stimulus beliefs are updated more instantly after positive outcomes and might underlie the stronger development of attentional capture for high-reward value.

We hypothesized the sensitivity of value-driven attention to be influenced by the weight that is put on learning from especially high-reward feedback. First, instrumental learning was directly followed by an attentional capture task in which participants searched for a shape singleton while a colored distractor was present on half the trials. The color of the distractors was the color most often receiving either a low or high reward in the learning task (see Fig. 1). Value-based attentional capture was expected to be strongest for colors previously associated with a high-value. Separate learning rates for high and low value were obtained by using a computational reinforcement-learning model (see Fig. 2) that reliably predicted individual trial-to-trial choices (see Fig. 3). High-value learning rates (*α*_*High*_) were expected to predict slowing with high-value distractors, whereas an explorative analysis focused on how learning from high- or low-value outcomes relates to the differential experience of high- and low-value distractors.

**Fig. 1:**
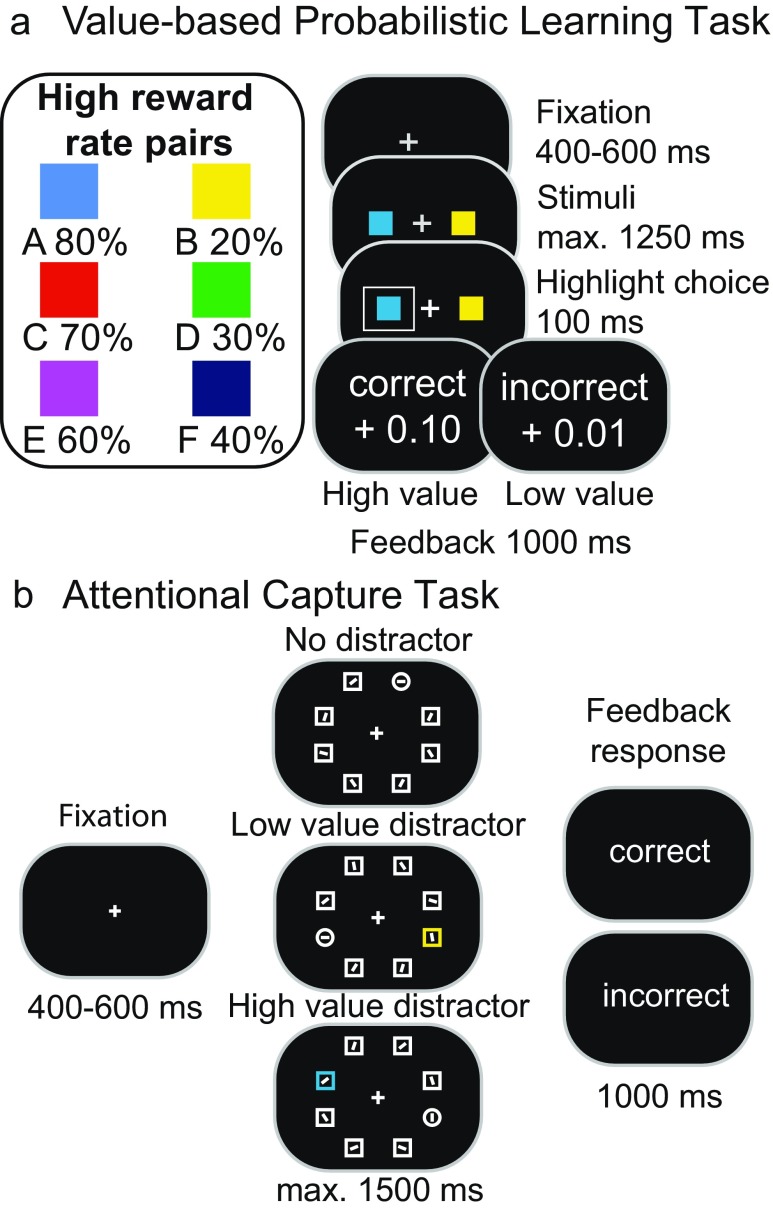
Illustration of both tasks. (a) Two colors were presented on each trial, and participants learned to select the colors that most often received a high reward feedback (A, C, E) solely through probabilistic feedback (probability of high reward is displayed beneath each stimulus). (b) Participants reported the orientation of the line segment within the shape singleton (the circle). On half the trials, one of the nontarget shapes was rendered in the color of the stimulus most often receiving low (B) or high (A) reward in the probabilistic learning task. (Color figure online)

**Fig. 2:**
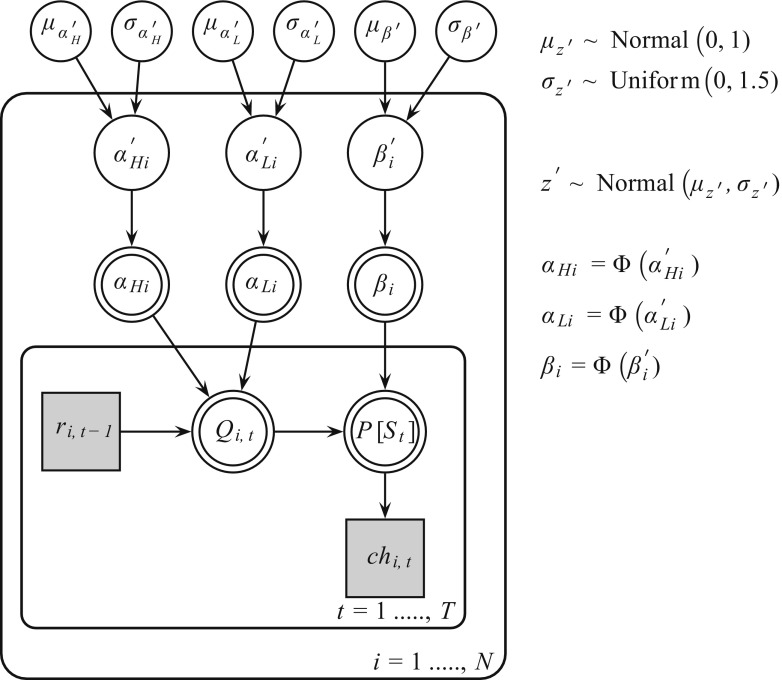
Bayesian graphical Q-learning model for hierarchical analysis. Φ () is the cumulative standard normal distribution function. The model consists of an outer subject (*i* = 1…,*N*), and an inner trial plane (*t* = 1,…,*T*). Nodes represent variables of interest. Arrows are used to indicate dependencies between variables. Double borders indicate deterministic variables. Continues variables are denoted with circular nodes, and discrete with square nodes. Observed variables are shaded in gray

**Fig. 3:**
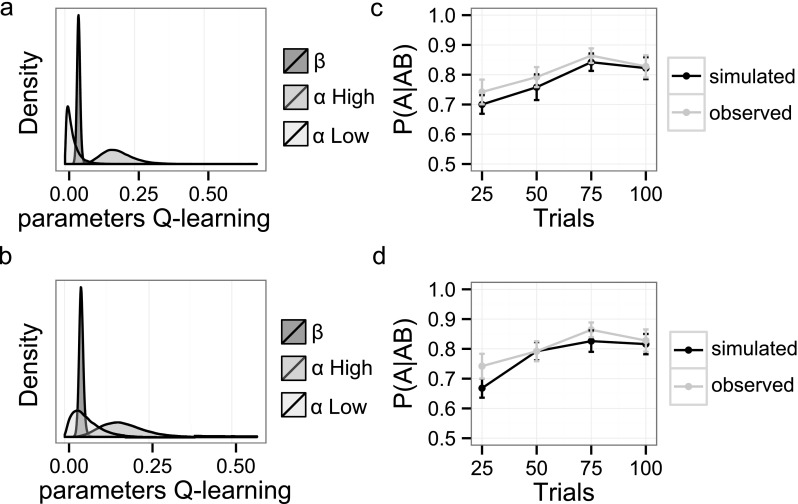
Posterior distributions and model evaluations. The left plane shows, group-level posteriors for all Q-learning parameters fit to either all choice options (a), or only to trials with choice option AB (b). In the right plane, the learning curve for choosing A over B, or P(A|AB), is simulated for each participant with the derived parameters and evaluated against the observed data for either fits to all choice options (c), or only AB trials (d). Error bars represent *SEM;* β/100 for visualization

## Method

### Participants

Twenty-one participants (six males, mean age = 23 years, range 18–31 years) with normal or corrected-to-normal vision participated for a monetary compensation (*M* = 11.5, *SD* = 0.3 euros). Sample-size was based on previous studies focusing on value-driven attentional capture (range = 16-26) (Anderson et al., 2011). One participant was excluded from all analyses because of chance-level performance. Informed consent was obtained from all participants, and the local ethics committee of the VU University Amsterdam approved all procedures.

### Procedure

The experiment was run on a calibrated 19-inch CRT monitor using OpenSesame (Mathôt, Schreij, & Theeuwes, 2012). Color–reward associations were obtained using the learning phase of a probabilistic learning task (Frank, Seeberger, & O’Reilly, 2004). Subsequently, an attentional capture task (Theeuwes, 1992) was presented to specify how learning influences attention (see Fig. 1).

### Value-based probabilistic learning

Three color pairs (AB, CD, EF) were presented in random order, and participants learned to choose one of the two color stimuli (see Fig. 1a). Colors were selected from a subset of six near-equiluminant colors (red, green, blue, yellow, purple, and turquoise), with an approximate luminance of 27.2 cd/m^2^ (*SD* = 5.2 cd/m^2^), and presented on a black background. For each participant, the pairs blue–yellow, red–green and purple–turquoise were randomly assigned to three categories (AB, CD, EF) and counterbalanced in mapping (e.g., blue–yellow or yellow–blue for AB). Probabilistic feedback followed each choice to indicate a high (“correct” +0.10 points) or low (“incorrect” +0.01 points) value. Choosing the high-value Color A lead to high rewards on 80% of the trials, whereas selecting the low-value Color B lead to low rewards with 80%. Other ratios for high reward were 70:30 (CD) and 60:40 (EF). Participants were told that the total sum of points earned would be transferred into a monetary reward at the end of the experiment. Trials started with a white fixation cross followed by two colored squares (1.67° × 1.67° visual angle) left and right of the fixation cross (2.1° distance to fixation). Choices were highlighted by a white frame (3.33° × 3.33° visual angle), and followed with feedback. Omissions or choices longer than 1,250 ms were followed with the text “too slow” for 300 ms. A 30-trial practice session was conducted to familiarize with the task (feedback: “correct” or “incorrect”) and followed by five blocks of 60 trials each (300 trials total; equal numbers of AB, CD and EF).

### Attentional capture

Participants searched for a unique circle shape (target) among five square shapes (distractors). Responses were based on the orientation of a vertical or horizontal line contained within the circle (see Fig. 1b). On half the trials, both target and distractors were presented in white (black background). For the other half, one of the distractor squares was rendered in the highly rewarded A-color or the low rewarded B-color. The target (circle) shape was always presented in white. Trials started with a white fixation cross followed by the search display. This display showed the fixation cross, surrounded by six shapes (1.67° × 1.67° visual angle) equally spaced along an imaginary circle (5.2° radius). Feedback indicated correct or incorrect responses. Participants started with a practice block of 20 trials, followed by 120 experiment trials.

### Reinforcement learning model: Q-learning

The influence of learning rates on attentional capture was investigated using the computational Q-learning algorithm (Daw, 2011; Frank et al., 2007; Watkins & Dayan, 1992). Because previous work has found stronger distractor effects for stimuli associated with high rewards, we defined separate learning rate parameters for high (*α*_*High*_) and low (*α*_*Low*_) value feedback (cf. Frank et al., 2007; Kahnt et al., 2009). Q-learning assumes participants will maintain reward expectation for each stimulus (A-to-F). The expected value (*Q*) for selecting a stimulus *i* (could be A-to-F) on the next trial is then updated as follows:

$$ {Q}_i\left(t+1\right)={Q}_i(t)+\left\{\begin{array}{l}{\alpha}_{High}\left[{r}_i(t)-{Q}_i(t)\right],\kern1em \mathrm{if}\;r=1\hfill \\ {}{\alpha}_{Low}\left[{r}_i(t)-{Q}_i(t)\right],\kern1em \mathrm{if}\;r=0\hfill \end{array}\right.\kern0.5em , $$

Where 0 ≤ *α*_*High / Low*_ ≤ 1 represent learning rates, *t* is trial number, and *r* = 1 (high) or *r* = 0 (low) reward. The probability of selecting one response over the other (i.e., A over B) is computed as:

$$ {P}_A(t)=\frac{ \exp \left(\beta \times {Q}_t(A)\right)}{ \exp \left(\beta \times {Q}_t(B)\right)+ \exp \left(\beta \times {Q}_t(A)\right)}\kern0.5em , $$

With 0 ≤ β ≤ 100 being known as the inverse temperature.

### Bayesian hierarchical estimation procedure

The Q-learning algorithm was fit using a Bayesian hierarchical estimation method where parameters for individual subjects are drawn from a group-level distribution. This hierarchical structure is preferred for parameter estimation because it allows for the simultaneous estimation of both group-level parameters and individual parameters (Lee, 2011; Steingroever, Wetzels, & Wagenmakers, 2013; Wetzels, Vandekerckhove, Tuerlinckx, & Wagenmakers, 2010).

Figure 2 shows a graphical representation of the model. The quantities *r*_*i*, *t*− 1_ (reward participant *i* on trial *t* - 1) and *ch*_*i*_, _*t*_ (choice participant *i* on trial *t*) can be obtained directly from the data. The quantities *α*_*Hi*_ (*α*_*High*_ participant *i*), *α*_*Li*_ (*α*_*Low*_ participant *i*) and *β*_*i*_ are deterministic because we model their respective probit transformations *z* ′ _*i*_ (*α*′_*Hi,*_ *α*′_*Li*_, *β*′_*i*_). The probit transform is the inverse cumulative distribution function of the normal distribution. The parameters *z* ′ _*i*_ lie on the probit scale covering the entire real line. Parameters *z* ′ _*i*_ were drawn from group-level normal distributions with mean *μ*_*z* ′_ and standard deviation *δ*_*z* ′_. A normal prior was assigned to group-level means ${\mu}_{z^{\prime }}\sim N\left(0,1\right)$, and a uniform prior to the group-level standard deviations ${\delta}_{z^{\prime }}\sim U\left(1,1.5\right)$ (Steingroever et al., 2013; Wetzels et al., 2010).

Two parallel versions of the Q-learning model were implemented to optimize fits to all trials (i.e., A-to-F), or only AB trials (used in the attention task). Both models were implemented in Stan (Homan & Gelman, 2014; Stan Development Team, 2014). Multiple chains were generated to ensure convergence, which was evaluated with the Rhat statistics (Gelman & Rubin, 1992). Evaluations ensured convergence for both fit procedures (i.e., all Rhats were close to 1). Figure 3 shows group-level posteriors (a, b), and data recovery evaluations (c, d).

The definition of two learning parameters was justified with the evaluation of a hierarchical Q-model with only one learning parameter, which was updated after each trial. Model selection was based on individual and group-level Bayesian Information Criterion (BIC), using a random-effects model on the log likelihoods (Jahfari, Waldorp, Ridderinkhof, & Scholte, 2015), and supported the use of two learning rates with lower BIC values (BIC_group: Q_2alpha = 2964, Q_1alpha = 3155; BIC_individual mean: Q_2alpha = 154, Q_1alpha = 162).

### Analysis

The choice for three probability pairs during training allowed us to compute and differentiate both specific (only considering the reliable 80–20 feedback) and general (across all contingencies) learning rates for high and low rewards. The choice for AB colors in the capture task was both pragmatic (considering the total number of distractor and nondistractor trials) and based on the literature, as value-based attention is commonly studied with a high reward contingency of 80%. Consistently, the attentional-capture task only used the most distinct A and B colors from the learning task (contingency 80–20). The relationship between value-based capture and learning parameters (*α*_*High*_ and *α*_*Low*_) was evaluated with model fits to (1) only AB trials or (2) all A-to-F trials. Because both learning parameters were restricted between 0 and 1, Spearman’s rank correlation (rho) or partial correlation (rho_pcor_) was used to evaluate capture–learning relationships.

## Results

### Value-based learning and attentional capture

In the learning task, subjects reliably learned to choose the most rewarded option from all three pairs. For each pair the probability of choosing the better option was above chance (*p*s < .001), and the effect of learning decreased from AB (*M* = 0.81, *SD* = 0.12) to CD (*M* = 0.73, *SD* = 0.17) to EF (*M* = 0.71, *SD* = 0.15), *F*(1, 19) = 5.12, *p* = .036, η^2par^ = 0.21.

**Fig. 4:**
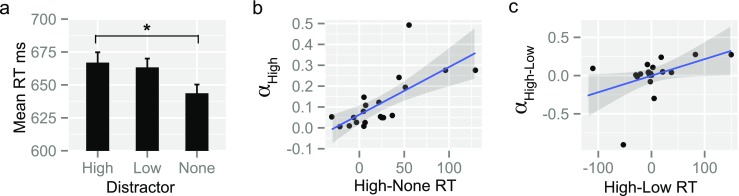
Individual differences in learning from high and low reward predict slowing in value-based capture. (a) Distractor colors consistently rewarded with high-value (High) slowed RT in comparison to no distractor trials (None), and the effect of learned value on attention was linear. Error bars reflect *SEM;* * = *p*(Bonferoni corrected). (b) Relationship between *α*_*High*_ and the magnitude of slowing caused by the high-value distractor. (c) Individual differences in learning from high- or low-value outcomes (α_High-Low_ = *α*_*High*_ – *α*_*Low*_) predicted RT differences between high- and low-value distractors

A repeated-measures ANOVA, with the factor distractor color (high-value, low-value, none) differentiated response times (RTs) across the three conditions, *F*(2, 38) = 3.36, *p* = .045, η^2par^ = 0.15. Distractor trials with a high-value color slowed RT in comparison to no-distractor trials, *t*(19) = 2.70, *p*(Bonferroni corrected) = .043, and the effects of value on performance was linear, *F*(1, 19) = 7.27, *p* = .014, η^2par^ = 0.28 (see Fig. 4a). However, a direct comparison of the low-value condition with either the high-value or no-distractor condition showed no reliable effects. Distractors had no effect on error rates, P(correct) high: *M*(*SD*) = 0.90(0.07), low: *M*(*SD*) = 0.90(0.07), None: *M*(*SD*) = 0.92(0.05), *p* = .181.

### Value-based attentional capture and learning rates

Next, we examined whether individual differences in the rate of information updating after desired (*α*_*High*_), or undesired (*α*_*Low*_) outcomes was predictive for the magnitude of automatic capture. Evaluations of the Q-learning model showed both fit-procedures to reliably predict individual trial-to-trial choices during the learning task (Fig. 3c,d). Learning parameters derived from the models where then used to examine the relationship with attentional capture (Table 1).

**Table 1:** Posterior modes of the estimated Q-learning parameters

	β	*α*_*High*_	*α*_*Low*_
Fit-to-AB	4.65 (0.97)	0.12 (0.12)	0.11 (0.26)
Fit-to-all	4.95 (1.55)	0.18 (0.18)	0.08 (0.20)

When the model was optimized to predict AB choices, results showed a strong relationship between *α*_*High*_ and high-value slowing (rho_pcor_ = 0.69, *p* = .00007; Fig. 4b), while controlling for the nonsignificant relationship between *α*_*Low*_ and high-value distractors (rho_pcor_ = 0.09, *p* = 0.70). No significant relationship was found between *α*_*High*_ and capture with low-value colors (*p* = .35). Hence, participants who updated their beliefs robustly after high rewards (higher *α*_*High*_) experienced more slowing when the distractor had the high-reward A color. This relationship was very specific to high-value learning rates and not predicted by the sampling/selection frequency of the A color (% correct AB pairs) during learning (rho = 0.24, *p* = .31), or the estimated belief (Q value A color) at the end of learning (rho = 0.24, *p* = .30). No relationship was found between learning rates and slowing when the model was optimized to predict all learning-task choices (A-to-F), with reward probabilities 80:20, 70:30, and 60:40 (all *p*s > .05).

Most nonreward studies find a significant slowing effect for colored singletons. However, attentional capture for low-reward colored singletons is not always found. We explored whether capture differences in RT between high- and low-reward distractors relate to learning differences in relation to high- or low-reward outcomes. Results showed larger differences between *α*_*High*_ and *α*_*Low*_ (α_H-L_ = *α*_*High*_ – *α*_*Low*_) to predict larger RT differences between high- and low-value distractors for AB-model fits (rho = 0.47, *p* = .04; Fig. 4c) and all trial model fits (rho = 0.59, *p* = .008). This relationship remained reliable after the removal of the lowest point for fits-to-all trials (rho = 0.52, *p* = .02), but was only marginal for fits-to-AB trials (rho = 0.40, *p* = .09).

## Discussion

This study relates the underlying mechanisms of reward learning to the development of value-based attentional capture. We showed how learning from high- or low-value outcomes develops into value-driven attentional biases. This finding sheds light on a surge of recent results focusing on the consequences of reward on attention. For example, value-driven capture is generally stronger when learning is based on high values (Anderson & Yantis, 2012; Chelazzi et al., 2013; Della Libera & Chelazzi, 2006). This has been attributed to the implicit assumption that high-value distractors capture attention more robustly than low-value distractors (Theeuwes & Belopolsky, 2012). We refine this assumption by demonstrating how individual differences in learning relate to the magnitude of value-driven attentional capture.

Our results show how value learning in a task that is completely unrelated to visual search may develop in robust value-driven capture. Such attentional biases were shown after classic conditioning, and instrumental tasks with a direct resemblance to the capture task (Anderson et al., 2011; Della Libera & Chelazzi, 2009; Hickey, Chelazzi, & Theeuwes, 2010), or a focus on next-trial decision modulations with previously rewarded distractors (Itthipuripat, Cha, Rangsipat, & Serences, 2015). We extend current beliefs by showing how instrumental learning can transfer into the automatic capture of attention for a single feature, irrespective of context (see Anderson, 2014, for differences with classic conditioning).

Attentional selection plays an important role during learning, and is especially useful if some information is more relevant (e.g., Dayan, Kakade, & Montague, 2000). Here, high- and low-value colors were always presented simultaneously during learning. Importantly, the subsequent capture task only showed reliable slowing effects for high-value colors. Neurophysiological work has suggested selective attention to suppress processing of undesired stimuli, which in effect may imply that only the high-reward stimulus is processed (Moran & Desimone, 1985). Optimal responses during learning could involve attentional priority toward the desired high-value color (leading to value-based capture), *and* suppression of the undesired low-value color (reduced distraction in future tasks).

Higher learning rates represent stronger trial-to-trial belief updates about the chosen stimuli and could motivate the advanced prioritization of the desired stimulus. This predicts participants with a steep learning rate (for high-value outcomes) to prioritize earlier and longer, and so experience more capture in future tasks (Kahnt, Park, Haynes, & Tobler, 2014; Störmer, Eppinger, & Li, 2014). Compatibly, we found belief updates after high-value outcomes to predict the degree of capture with high-value distractors. A final explorative analysis indicated how learning rate differences from high- and low-value outcomes relate to capture differences, given a low- or high-value distractor. Participants who learned faster from positive outcomes, experienced more capture from high- than from low-value distractors. These findings indicate learning rates to modulate selective attention during learning, and by doing so, shape the experience of capture in future contexts.

Notably, the transfer of value into capture was sensitive to both high value and feedback consistency. However, the differential capture of attention with high- or low-reward distractors was more sensitive to how we learn differentially from reward magnitude in general. These probability specific (i.e., transfer) and general (i.e., differential experience) relationships are novel and should be studied further to understand the significance of either magnitude, or consistency, in the development of automatic attention. For example, future designs could use only high-value distractors, while feedback consistency is varied during learning (O’Doherty, 2014).

This study provides novel prospects to incorporate both computational and neuroscience theories in our understanding of value-driven capture. For example, the magnitude of learning from positive feedback is attributed to striatal dopamine levels, whereas trial-to-trial adjustments after a single instance of negative feedback relate to elevated dopamine within the prefrontal cortex (PFC; Frank et al., 2007). PFC learning effects are part of a controlled learning system with a strong dependence on working memory capacity (Collins & Frank, 2012), and increased dopamine levels within PFC are reported to overstabilize working memory representations such that they persist over time (Durstewitz, Seamans, & Sejnowski, 2000). The effects found with high-value rewards could rely on higher dopamine levels within the striatum, a region not restricted by memory decay or capacity and central to the formation of “habit memory” (Knowlton, Mangels, & Squire, 1996; Pasupathy & Miller, 2005). Consistently, elevated levels of dopamine in PFC could selectively modulate the less intuitive learning rates after low-value outcomes through the stabilization of working memory representations, and so influence their transfer into future capture. High-value capture is recently linked to working memory performances (Anderson et al., 2011), prediction (Sali, Anderson, & Yantis, 2014), and dopamine (Anderson et al., 2016; Hickey & Peelen, 2015). Future couplings between dopaminergic learning and the formation of automatic attention can have substantial implications for patient populations such as those with Parkinson’s or attention-deficit/hyperactivity disorders.
